# 1-(5-Bromo-2-hydr­oxy-4-methoxy­phen­yl)ethanone

**DOI:** 10.1107/S1600536809029067

**Published:** 2009-07-25

**Authors:** Wei-Xia Qing, Yan-Zhao Liu, Su-Mei Yao

**Affiliations:** a Medical College of Henan University, Henan University, Kaifeng 475004, People’s Republic of China; b Henan Quality Polytechnic, Pingdingshan, 467000, People’s Republic of China

## Abstract

In the title compound, C_9_H_9_BrO_3_, the dihedral angle between the ethanone group and the aromatic ring is 3.6 (2)°. The mol­ecular conformation is consolidated by an intra­molecular O—H⋯O hydrogen bond. The crystal structure is stabilized by π–π inter­actions between the benzene rings [centroid–centroid distance = 3.588 (2) Å].

## Related literature

1-(5-Bromo-2-hydr­oxy-4-methoxy­phen­yl)ethanone is one of the main components of the traditional Chinese medicine Moutan Cortex, which is also a valuable spice and is widely used in domestic chemistry, see: Chung (1999 ▶); Liu *et al.* (2000 ▶). For our work on the preparation of derivatives, see: Qi *et al.* (2003 ▶).
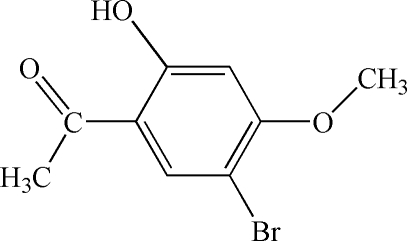


## Experimental

### 

#### Crystal data

C_9_H_9_BrO_3_
*M* *_r_* = 245.07
Monoclinic, 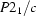

*a* = 9.916 (3) Å
*b* = 13.836 (5) Å
*c* = 6.940 (2) Å
β = 90.031 (3)°
*V* = 952.0 (5) Å^3^
*Z* = 4
Mo *K*α radiation
μ = 4.29 mm^−1^
*T* = 296 K
0.24 × 0.13 × 0.09 mm

#### Data collection

Bruker SMART CCD area-detector diffractometer
Absorption correction: multi-scan (*SADABS*; Sheldrick, 2001 ▶) *T*_min_ = 0.426, *T*_max_ = 0.699
5163 measured reflections
1860 independent reflections
977 reflections with *I* > 2σ(*I*)
*R*_int_ = 0.080

#### Refinement

*R*[*F* ^2^ > 2σ(*F* ^2^)] = 0.045
*wR*(*F* ^2^) = 0.068
*S* = 1.01
1860 reflections
118 parameters
H-atom parameters constrained
Δρ_max_ = 0.46 e Å^−3^
Δρ_min_ = −0.52 e Å^−3^

### 

Data collection: *SMART* (Bruker, 2001 ▶); cell refinement: *SAINT-Plus* (Bruker, 2001 ▶); data reduction: *SAINT-Plus*; program(s) used to solve structure: *SHELXS97* (Sheldrick, 2008 ▶); program(s) used to refine structure: *SHELXL97* (Sheldrick, 2008 ▶); molecular graphics: *PLATON* (Spek, 2009 ▶); software used to prepare material for publication: *PLATON*.

## Supplementary Material

Crystal structure: contains datablocks global, I. DOI: 10.1107/S1600536809029067/at2849sup1.cif

Structure factors: contains datablocks I. DOI: 10.1107/S1600536809029067/at2849Isup2.hkl

Additional supplementary materials:  crystallographic information; 3D view; checkCIF report

## Figures and Tables

**Table 1:** Hydrogen-bond geometry (Å, °)

*D*—H⋯*A*	*D*—H	H⋯*A*	*D*⋯*A*	*D*—H⋯*A*
O1—H1*A*⋯O3	0.82	1.83	2.549 (4)	146

**Fig. 1.**
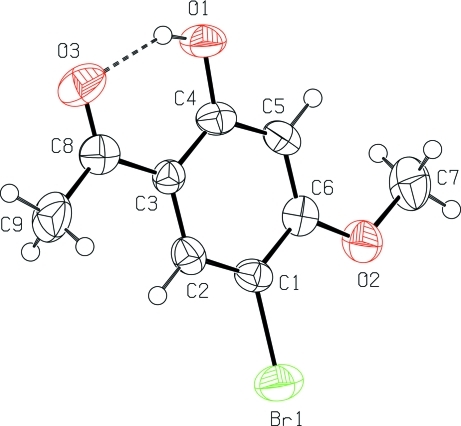
The molecular structure of (I), showing the atom-labelling scheme. Displacement ellipsoids are drawn at the 50% probability level. An intramolecular O—H···O hydrogen bond is indicated by the dashed line.

## supplementary crystallographic information

### Comment

1-(5-Bromo-2-hydroxy-4-methoxyphenyl)ethanone is one of the main components of traditional Chinese medicine Moutan Cortex, which is also a valuable inartificial spicery and can be widely used in domestic chemistry (Chung, 1999; Liu, *et al.* 2000). But the nature of water insolubility and volatility makes it difficult to exert its efficiency sufficiently. Preparing derivatives has been an active research area (Qi, *et al.* 2003) for a long time. Herein we report the crystal structure of the title compound (I).

Compound (I) consists of an asymmetric organic molecule (Fig.1). The C1—C6 benzene ring in (I) is an aromatic ring, on which four different organic groups decorated. In the structure, C8—O3 [1.224 (4) Å] is typical for a C═O double bond, whereas, the C4—O1, C6—O2 and C7—O2 bond distances are of 1.347 (4), 1.351 (4) and 1.420 (4) Å, respectively, indicating three obviously C—O single bonds.

In addition, the intramolecular hydrogen bond exhibit in the compound, O1—H1A acting as hydrogen bond donor, and O3 atom as hydrogen bond acceptor, constructing a S(6) ring (Fig.1, Table 1). The crystal structure is stabilized by π-π interactions between the benzene rings [centroid-to-centroid distance = 3.588 (2) Å].

### Experimental

2-Hydroxyl-4-methoxyacetophenone was isolated from the Chinese medicine Moutan Cortex. *N*-Bromosuccinimide (0.534 g, 3 mmol) was added slowly by cannulation to a stirred suspension of 2-hydroxyl-4-methoxyacetophenone (0.499 g, 3 mmol) in chloroform (50 ml) at room temperature. After stirring for 1 h the solution was quenched with saturated aqueous sodium bicarbonate solution (20 ml) the layers were separated and the aqueous layer was extracted with chloroform, the combined organic extracts were washed with water (20 ml), dried (MgSO_4_) and evaporated under reduced pressure to give the crude product. Then purification by short column chromatography (chloroform) and recrystallization from chloroform gave the compound (I) as needle-like colourless crystal (0.645 g, 88%).

### Refinement

H atoms were treated as riding, with C—H distances of 0.93 Å–0.96 Å and O—H distances of 0.82 Å, and were refined as riding with *U*_iso_(*H*) = 1.2*U*_eq_(C in aromatic ring) and *U*_iso_(*H*) = 1.5*U*_eq_(O or C_methyl_).

### Crystal data

**Table:**

C_9_H_9_BrO_3_	*F*(000) = 488
*M**_r_* = 245.07	*D*_x_ = 1.710 Mg m^−^^3^
Monoclinic, *P*2_1_/*c*	Mo *K*α radiation, λ = 0.71073 Å
Hall symbol: -P 2ybc	Cell parameters from 1164 reflections
*a* = 9.916 (3) Å	θ = 2.5–21.4°
*b* = 13.836 (5) Å	µ = 4.29 mm^−^^1^
*c* = 6.940 (2) Å	*T* = 296 K
β = 90.031 (3)°	Needle-like, colourless
*V* = 952.0 (5) Å^3^	0.24 × 0.13 × 0.09 mm
*Z* = 4	

### Data collection

**Table:**

Bruker SMART CCD area-detector diffractometer	1860 independent reflections
Radiation source: fine-focus sealed tube	977 reflections with *I* > 2σ(*I*)
graphite	*R*_int_ = 0.080
φ and ω scans	θ_max_ = 26.0°, θ_min_ = 2.1°
Absorption correction: multi-scan (*SADABS*; Sheldrick, 2001)	*h* = −12→9
*T*_min_ = 0.426, *T*_max_ = 0.699	*k* = −17→16
5163 measured reflections	*l* = −8→8

### Refinement

**Table:**

Refinement on *F*^2^	Primary atom site location: structure-invariant direct methods
Least-squares matrix: full	Secondary atom site location: difference Fourier map
*R*[*F*^2^ > 2σ(*F*^2^)] = 0.045	Hydrogen site location: inferred from neighbouring sites
*wR*(*F*^2^) = 0.068	H-atom parameters constrained
*S* = 1.01	*w* = 1/[σ^2^(*F*_o_^2^) + (0.001*P*)^2^] where *P* = (*F*_o_^2^ + 2*F*_c_^2^)/3
1860 reflections	(Δ/σ)_max_ = 0.001
118 parameters	Δρ_max_ = 0.46 e Å^−^^3^
0 restraints	Δρ_min_ = −0.52 e Å^−^^3^

### Special details

**Table:**

Geometry. All e.s.d.'s (except the e.s.d. in the dihedral angle between two l.s. planes) are estimated using the full covariance matrix. The cell e.s.d.'s are taken into account individually in the estimation of e.s.d.'s in distances, angles and torsion angles; correlations between e.s.d.'s in cell parameters are only used when they are defined by crystal symmetry. An approximate (isotropic) treatment of cell e.s.d.'s is used for estimating e.s.d.'s involving l.s. planes.
Refinement. Refinement of *F*^2^ against ALL reflections. The weighted *R*-factor *wR* and goodness of fit *S* are based on *F*^2^, conventional *R*-factors *R* are based on *F*, with *F* set to zero for negative *F*^2^. The threshold expression of *F*^2^ > σ(*F*^2^) is used only for calculating *R*-factors(gt) *etc*. and is not relevant to the choice of reflections for refinement. *R*-factors based on *F*^2^ are statistically about twice as large as those based on *F*, and *R*- factors based on ALL data will be even larger.

### Fractional atomic coordinates and isotropic or equivalent isotropic displacement parameters (Å^2^)

**Table:**

	*x*	*y*	*z*	*U*_iso_*/*U*_eq_	
Br1	0.67087 (5)	0.52356 (3)	0.17229 (7)	0.0635 (2)	
O1	1.0051 (3)	0.88085 (17)	0.2857 (4)	0.0522 (8)	
H1A	1.0839	0.8682	0.3109	0.078*	
O2	0.5841 (3)	0.7279 (2)	0.1634 (4)	0.0514 (8)	
O3	1.2075 (3)	0.7721 (2)	0.3509 (4)	0.0625 (9)	
C1	0.7770 (4)	0.6352 (3)	0.2110 (5)	0.0377 (11)	
C2	0.9133 (4)	0.6264 (3)	0.2495 (5)	0.0398 (11)	
H2A	0.9515	0.5651	0.2572	0.048*	
C3	0.9948 (4)	0.7076 (3)	0.2771 (5)	0.0336 (10)	
C4	0.9349 (4)	0.7981 (3)	0.2651 (5)	0.0379 (11)	
C5	0.7969 (4)	0.8080 (3)	0.2272 (5)	0.0393 (11)	
H5A	0.7584	0.8692	0.2200	0.047*	
C6	0.7179 (4)	0.7270 (3)	0.2006 (5)	0.0382 (11)	
C7	0.5177 (4)	0.8188 (3)	0.1589 (7)	0.0638 (14)	
H7A	0.4238	0.8093	0.1308	0.096*	
H7B	0.5575	0.8586	0.0610	0.096*	
H7C	0.5269	0.8499	0.2819	0.096*	
C8	1.1410 (5)	0.6994 (3)	0.3182 (6)	0.0445 (12)	
C9	1.2063 (4)	0.6027 (3)	0.3192 (7)	0.0649 (14)	
H9A	1.3005	0.6097	0.3482	0.097*	
H9B	1.1962	0.5732	0.1948	0.097*	
H9C	1.1645	0.5627	0.4151	0.097*	

### Atomic displacement parameters (Å^2^)

**Table:**

	*U*^11^	*U*^22^	*U*^33^	*U*^12^	*U*^13^	*U*^23^
Br1	0.0606 (3)	0.0477 (3)	0.0823 (4)	−0.0137 (3)	−0.0044 (3)	−0.0034 (3)
O1	0.051 (2)	0.0372 (19)	0.069 (2)	−0.0123 (15)	0.0022 (16)	0.0005 (16)
O2	0.038 (2)	0.051 (2)	0.065 (2)	0.0066 (16)	−0.0004 (16)	0.0018 (16)
O3	0.043 (2)	0.068 (2)	0.077 (2)	−0.0145 (17)	−0.0017 (17)	−0.0097 (19)
C1	0.045 (3)	0.033 (3)	0.036 (3)	−0.003 (2)	0.005 (2)	0.002 (2)
C2	0.043 (3)	0.038 (3)	0.038 (3)	0.008 (2)	0.000 (2)	0.001 (2)
C3	0.035 (3)	0.035 (3)	0.030 (3)	0.001 (2)	−0.001 (2)	−0.002 (2)
C4	0.044 (3)	0.037 (3)	0.033 (3)	−0.009 (2)	0.010 (2)	0.001 (2)
C5	0.051 (3)	0.030 (3)	0.037 (3)	0.004 (2)	0.004 (2)	0.003 (2)
C6	0.035 (3)	0.048 (3)	0.031 (3)	0.003 (2)	0.007 (2)	−0.002 (2)
C7	0.037 (3)	0.081 (4)	0.074 (4)	0.015 (3)	0.001 (3)	0.008 (3)
C8	0.049 (3)	0.048 (3)	0.036 (3)	0.000 (3)	0.007 (2)	−0.004 (2)
C9	0.041 (3)	0.076 (4)	0.078 (4)	0.009 (3)	−0.016 (3)	−0.006 (3)

### Geometric parameters (Å, °)

**Table:**

Br1—C1	1.889 (4)	C3—C8	1.482 (5)
O1—C4	1.347 (4)	C4—C5	1.400 (5)
O1—H1A	0.8200	C5—C6	1.380 (5)
O2—C6	1.351 (4)	C5—H5A	0.9300
O2—C7	1.420 (4)	C7—H7A	0.9600
O3—C8	1.224 (4)	C7—H7B	0.9600
C1—C2	1.382 (5)	C7—H7C	0.9600
C1—C6	1.400 (5)	C8—C9	1.487 (5)
C2—C3	1.397 (5)	C9—H9A	0.9600
C2—H2A	0.9300	C9—H9B	0.9600
C3—C4	1.389 (5)	C9—H9C	0.9600
			
C4—O1—H1A	109.5	O2—C6—C1	115.5 (4)
C6—O2—C7	117.9 (3)	C5—C6—C1	119.4 (4)
C2—C1—C6	120.0 (4)	O2—C7—H7A	109.5
C2—C1—Br1	120.0 (3)	O2—C7—H7B	109.5
C6—C1—Br1	120.0 (3)	H7A—C7—H7B	109.5
C1—C2—C3	121.4 (4)	O2—C7—H7C	109.5
C1—C2—H2A	119.3	H7A—C7—H7C	109.5
C3—C2—H2A	119.3	H7B—C7—H7C	109.5
C4—C3—C2	118.0 (4)	O3—C8—C3	120.0 (4)
C4—C3—C8	119.9 (4)	O3—C8—C9	120.3 (4)
C2—C3—C8	122.1 (4)	C3—C8—C9	119.7 (4)
O1—C4—C3	122.6 (4)	C8—C9—H9A	109.5
O1—C4—C5	116.2 (4)	C8—C9—H9B	109.5
C3—C4—C5	121.1 (4)	H9A—C9—H9B	109.5
C6—C5—C4	120.0 (4)	C8—C9—H9C	109.5
C6—C5—H5A	120.0	H9A—C9—H9C	109.5
C4—C5—H5A	120.0	H9B—C9—H9C	109.5
O2—C6—C5	125.1 (4)		
			
C6—C1—C2—C3	−0.5 (6)	C7—O2—C6—C1	177.5 (4)
Br1—C1—C2—C3	179.4 (3)	C4—C5—C6—O2	180.0 (3)
C1—C2—C3—C4	0.1 (6)	C4—C5—C6—C1	−0.3 (6)
C1—C2—C3—C8	−179.9 (3)	C2—C1—C6—O2	−179.6 (3)
C2—C3—C4—O1	−178.7 (4)	Br1—C1—C6—O2	0.4 (5)
C8—C3—C4—O1	1.4 (5)	C2—C1—C6—C5	0.6 (6)
C2—C3—C4—C5	0.2 (5)	Br1—C1—C6—C5	−179.3 (3)
C8—C3—C4—C5	−179.8 (3)	C4—C3—C8—O3	3.6 (6)
O1—C4—C5—C6	178.8 (3)	C2—C3—C8—O3	−176.4 (4)
C3—C4—C5—C6	−0.1 (6)	C4—C3—C8—C9	−176.3 (4)
C7—O2—C6—C5	−2.7 (5)	C2—C3—C8—C9	3.7 (5)

### Hydrogen-bond geometry (Å, °)

**Table:**

*D*—H···*A*	*D*—H	H···*A*	*D*···*A*	*D*—H···*A*
O1—H1A···O3	0.82	1.83	2.549 (4)	146
